# Cognitive Impairment in Cardiovascular Patients after Myocardial Infarction: Prospective Clinical Study

**DOI:** 10.3390/jcm12154954

**Published:** 2023-07-27

**Authors:** Dominika Kasprzak, Katarzyna Kaczmarek-Majer, Janusz Rzeźniczak, Katarzyna Klamecka-Pohl, Teresa Ganowicz-Kaatz, Marek Słomczyński, Jan Budzianowski, Konrad Pieszko, Jarosław Hiczkiewicz, Andrzej Tykarski, Paweł Burchardt

**Affiliations:** 1 Department of Cardiology, J. Strus Hospital, 61-285 Poznań, Poland; 2 Stochastic Methods Department, System Research Institute, Polish Academy of Sciences, 01-447 Warsaw, Poland; 3 Analyx Sp. z o.o. sp.k., 61-887 Poznań, Poland; 4 Faculty of Medicine and Health Sciences, University of Zielona Góra, 65-417 Zielona Góra, Poland; 5 Department of Hypertension, Angiology, and Internal Medicine, Poznan University of Medical Sciences, 61-848 Poznań, Poland

**Keywords:** acute coronary syndrome, cognitive impairment, dementia, mental disorders, myocardial infarction

## Abstract

(1) Background: Assessment of cognitive function is not routine in cardiac patients, and knowledge on the subject remains limited. The aim of this study was to assess post-myocardial infarction (MI) cognitive functioning in order to determine the frequency of cognitive impairment (CI) and to identify factors that may influence it. (2) Methods: A prospective study included 468 patients hospitalized for MI. Participants were assessed twice: during the first hospitalization and 6 months later. The Mini-Mental State Examination was used to assess the occurrence of CI. (3) Results: Cognitive dysfunction based on the MMSE was found in 37% (N-174) of patients during the first hospitalization. After 6 months, the prevalence of deficits decreased significantly to 25% (N-91) (*p* < 0.001). Patients with CI significantly differed from those without peri-infarction deficits in the GFR, BNP, ejection fraction and SYNTAX score, while after 6 months, significant differences were observed in LDL and HCT levels. There was a high prevalence of non-cognitive mental disorders among post-MI patients. (4) Conclusions: There is a high prevalence of CI and other non-cognitive mental disorders, such as depression, sleep disorders and a tendency to aggression, among post-MI patients. The analysis of the collected material indicates a significant impact of worse cardiac function expressed as EF and BNP, greater severity of coronary atherosclerosis expressed by SYNTAX results, and red blood cell parameters and LDL levels on the occurrence of CI in the post-MI patient population.

## 1. Introduction

Coronary heart disease (CHD) with its most common manifestation, myocardial infarction (MI), is a leading cause of morbidity and mortality worldwide [1]. At the same time, mainly due to an aging population, there is a growing problem of cognitive impairment (CI) and dementia. It is estimated that approximately 50 million of the world’s population has dementia, and the WHO predicts that by 2030, this number will have reached 82 million [2]. Patients presenting with CI that does not impair their daily functioning meet the criteria for the diagnosis of mild cognitive impairment (MCI). However, it is associated with an increased risk of developing dementia, with an annual progression rate of between 5 and 10% [3]. 

There is growing evidence suggesting an association between CHD and the occurrence of MCI and dementia [4,5], while the direct impact of MI on cognitive function remains unclear, even controversial. Research on cognitive function remains a challenge for researchers due to the high heterogeneity of these disorders both in terms of etiology and the tests used to assess them. Consequently, the exact mechanisms responsible for the development of MCI and dementia in the CHD patient population remain largely unexplored. Among others, they share common atherosclerotic risk factors, such as obesity, diabetes, hypertension, hypercholesterolemia and physical inactivity [6]. When cardiovascular risk factors and cognitive deficits are present, vascular cognitive impairment (the second most common cause of dementia after Alzheimer’s disease) is most often suspected. Despite this, post hoc meta-regression analyses conducted in the CHD patient population showed no difference between studies that corrected for cardiovascular risk factors and those that did not. This suggests that the relationship between CHD and dementia is more complex and cannot be explained solely by sharing common atherosclerotic risk factors [5]. 

To date, a limited number of studies attempting an assessment of cognitive function in patients after MI have been published. Moreover, their assessment is also not part of routine daily clinical practice among cardiologists [7]. This may be due to the low awareness of the widespread presence of MCI and dementia in their patient population and a focus mainly on cardiovascular problems, neglecting a holistic approach to the patient. However, available data indicate that CHD increases the risk of MCI by 45% [5], and acute coronary syndromes (ACSs) are associated with their faster progression [8]. Moreover, the presence of cognitive deficits especially in post-MI patients carries serious clinical implications. As it is associated with poorer compliance and adherence to medical recommendations, it may make it more difficult for patients to achieve secondary prevention goals, crucial in preventing further coronary incidents [9,10]. It is comforting to note that awareness of the link between mental disorders and cardiovascular disease (CVD) is also growing among cardiologists, as reflected in recent cardiovascular prevention guidelines addressing this issue [11].

Given these implications, we decided to undertake an assessment of the prevalence of CI in patients with MI and 6 months later and identify factors influencing it. In order to conduct a comprehensive analysis of the mental state of patients after MI, they were also assessed for the presence of other psychiatric disorders including depressive disorders, sleep disorders and aggressive behavior. 

## 2. Materials and Methods

### 2.1. Prospective Multicentral Study

The study was of a prospective multicenter type. Its protocol was approved by the Local Bioethics Committee (Agreement no. 1201/16). All participants were informed about the procedures and gave written informed consent to participate. Analyses were based on data from 468 patients hospitalized for MI (STEMI and NSTEMI), treated by percutaneous coronary intervention (PCI), who were then followed up for 6 months. Patients with a documented diagnosis of dementia, those not consenting to participate in the study and those unable to complete the study instruments independently were excluded. Using available medical records, a questionnaire on the patient’s health history, the SYNTAX score to assess the severity and complexity of coronary artery disease and laboratory and echocardiographic examination data were obtained on the baseline health status of the study participants. The same tests were performed after 6 months.

### 2.2. Psychiatric Assessment of Cognitive Function and Other Mental Disorders

Participants underwent two assessments of cognitive function: during the first MI-related hospitalization (on day 2 or 3 after coronary revascularization) and 6 months later. The Mini-Mental State Examination (MMSE) was used for this purpose. The MMSE assesses 6 cognitive domains: orientation, registration, attention and calculation, recall, language function and constructional praxis. The maximum score possible is 30, while a score <27 supports the presence of MCI or dementia syndrome. In order to fully assess mental status, patients were also assessed using the Beck Depression Inventory (BDI), the Modified Overt Aggression Scale (OAS-M), the Athens Insomnia Scale (AIS), the Epworth Sleepiness Scale (ES) and the Insomnia Severity Index (ISI). A single, appropriately trained person was engaged to administer all the aforementioned tests. The tests were performed according to the authors’ recommendations. In the case of the BDI, a score of ≥12 was established as the cut-off point indicating the presence of depressive symptoms. Insomnia was diagnosed in patients who scored 6 points and above on the AIS or ≥8 points on the ISI. A score of 11 and above on the ES was established as the threshold for sleepiness. For the OAS-M, only a score of 0 was considered normal, indicating no tendency toward aggression.

### 2.3. Statistical Methods

Statistical analyses were performed using the R language. Continuous variables were reported as means (standard deviation (SD)) or medians (interquartile ranges (IQR)), as appropriate. Qualitative data were presented as number and/or percentage. We consider the following two groups of patients depending on the occurrence of CI: (1) patients with CI; (2) without CI. Normal distribution of continuous variables was tested using the Shapiro–Wilk test. Next, the Mann–Whitney test was used for not normally distributed variables, and Student’s *t*-test was used for normally distributed variables. For the categorical variables, the Pearson chi square test for independence was applied. Generalized linear mixed-effects models were applied to quantify the strength of the relation between the clinical data collected during both visits and the intensity of various symptoms based on the psychiatric assessments for a given patient. Multivariate tables were performed for dichotomous variables, and McNemar’s test for paired nominal data was applied. Statistical significance was set at *p* = 0.05.

## 3. Results

### 3.1. Study Sample

A total of 468 patients hospitalized for MI (STEMI and NSTEMI) treated by percutaneous coronary intervention (PCI) were included in the study. Of these, 364 patients were reassessed for cognitive status after 6 months. The reason for the lower number of patients included in the follow-up was the outbreak of the COVID-19 pandemic, preventing follow-up visits for some study participants. The mean age was 59 (9.6) years, with men comprising 76% (N-358) of the group. Sixty percent of patients had a diagnosis of ACS STEMI (N-281). The subjects were characterized as overweight, with a median BMI of 27.8 (25–31.2) kg/m^2^. Twenty percent (N-95) of the study population had a history of diabetes, 58% (N-273) hypertension and 10% (N-46) heart failure. In addition, 12% (N-56) had experienced an MI.

### 3.2. Cognitive Impairment

Cognitive dysfunction based on the MMSE was found in 37% (N-174) of patients during the first hospitalization. After 6 months, the prevalence of deficits decreased significantly to 25% (N-91) (*p* < 0.001). A total of 15.7% of patients (N = 57) presented with sustained CI (both in the peri-infarct period and after 6 months). In 20.7% (N = 75) of study participants, deficits were found only in the peri-infarct period, while 8.8% (N = 32) presented only after 6 months.

For further analysis, patients were divided into two groups: with and without CI. We analyzed whether we could observe statistically significant differences between groups for both visits separately. Table 1 and Table 2 present a comparison of patients with cognitive disorders and without during the first hospitalization and follow-up.

As indicated in Table 1, patients with CI found during hospitalization for MI had significantly lower GFRs (85 (73–101) vs. 90 (76–105); *p* = 0.02), as well as higher BNP levels (127.4 (73.1–265.1) vs. 96.9 (53.2–199.8); *p* = 0.01) and lower ejection fraction (48 (41–50) vs. 48 (45–54); *p* = 0.05), compared to those without deficits. It was also observed that those with CI were significantly older (60.4 (±10.2) vs. 58.3 (±9.3); *p* = 0.03) and scored higher on the SYNTAX scale (10 (7–16) vs. 9 (6–14); *p* = 0.05).

As indicated in Table 2, at a 6-month follow-up, it was observed that those with CI compared to those without deficits had significantly higher LDL levels (2.1 (1.6–2.6) vs. 1.8 (1.5–2.3); *p* = 0.05) and HDL levels (1.4 (1.1–1.6) vs. 1.2 (1–1.5); *p* = 0.05), as well as lower HCT (42.3 (±3.3) vs. 43.1 (±3.5); *p* = 0.02).

### 3.3. Relationship between Severity of CI and Clinical Parameters

In the next step, the relationships between clinical parameters and the severity of CI expressed as total MMSE scores were assessed. The results show that the greater the severity of cognitive deficits (a lower MMSE score), (1) the lower the hemoglobin level (*p* < 0.001), (2) the lower the hematocrit level (*p* < 0.001), (3) the lower the erythrocyte level (<0.001) and (4) the higher the LDL level (<0.001). The results are presented in Table 3.

### 3.4. Other Mental Disorders and Cognitive Deficits

A high prevalence of mental status disorders other than cognitive disorders was found among patients after MI. The prevalence of depression, sleep disorders (insomnia and excessive sleepiness) and a tendency to aggression in the peri-infarct period and after 6 months is shown in Table 4. All disorders, except aggression and excessive sleepiness, were significantly more common during the first MI-related hospitalization.

Analysis of the relationship between the severity of selected psychiatric disorders and cognitive function showed that patients with greater CI severity (a lower MMSE score) were characterized by (1) greater severity of insomnia (expressed by higher scores on the ISI scale) and (2) greater tendency to aggression (expressed by the sum of scores obtained on the aggression scale). For aggression, the correlation was only observed in the female group. The results are presented in Table 5.

In addition, as indicated in Table 1, patients with CI found during their first hospitalization scored significantly higher on the Beck Depression Scale (9 (6–14) vs. 7 (4–13), *p* = 0.02), Athens Insomnia Scale (7 (3–10) vs. 5 (3–8); *p* = 0.04) and ISI scale (9 (4–14) vs. 7 (3–11); *p* < 0.001) compared to those without the disorder.

## 4. Discussion

Little is known about the prevalence of CI in patients after MI. The studies conducted to date are characterized by wide variability in the screening tests used and the diagnostic criteria, which translates into a high variability in the prevalence of CI in this group of patients ranging from 9 to 85% [12]. 

Our results show the prevalence of cognitive deficits in patients hospitalized for MI. CI of varying degrees of severity was found in about 40% of study participants. During the 6-month follow-up, some of them regressed, while they appeared de novo in a small group of patients. However, data from clinical trials indicate a higher risk of progression to dementia also among those whose cognitive status returned to normal. We are currently unable to predict the direction of these changes [13]. The presence of cognitive dysfunctions in patients after MI is significantly associated with difficulties in complying with medical recommendations and therefore in achieving the goals of secondary prevention. As a consequence, these people are at a higher risk of another heart attack, re-hospitalization and higher treatment costs [11]. For this reason, it is important to be aware of the prevalence of CI in this group of patients and to know the factors predisposing to them.

In our study, patients with CI in the peri-infarction period were characterized by significantly worse cardiac function, expressed in the laboratory by a higher level of BNP and in echocardiography by a lower ejection fraction. Previous studies have also linked heart failure with CI and dementia. Possible mechanisms point to impaired cerebral blood flow with reduced cardiac output [14]. Publications in the last few years have also reported a link between BNP levels and the development of dementia. In these observations, BNP appears as a marker of cardiac dysfunction underlying subclinical brain damage and, consequently, dementia [15].

We also observed that patients presenting with peri-infarct CI scored significantly higher on the SYNTAX scale assessing the complexity of atherosclerotic lesions in the coronary arteries. A recent study showed that the SYNTAX result may be a predictor of clinically silent central nervous system embolization after coronary angiography and PCI [16]. Therefore, it would seem that individuals with higher SYNTAX results could be subject to closer scrutiny regarding their cognitive status.

Among the factors influencing the occurrence of CI, the role of hypercholesterolemia should be highlighted. Our results confirm the findings of other studies as well and indicate an association between higher LDL cholesterol levels and greater severity of neurocognitive impairment [17]. Six months after MI, patients without cognitive deficits had significantly lower LDL levels than those with CI. Low LDL cholesterol is important for secondary prevention and preventing another coronary incident. However, our results show that it may also have an impact on cognitive abilities, crucial for the quality of life, and this may be of great importance for patients. Interpreting them, however, raises the question regarding the impact of hypolipemic treatment on cognitive function. To fully understand the impact of dyslipidemia on these functions, the results should be viewed from the perspective of the treatment used. Although the patients included in our study received intensive hypolipemic treatment, pharmacotherapy was not the focus of the present study and is an area for further analysis. We also obtained inconclusive results in the context of HDL cholesterol levels, which were found to be significantly higher in patients with CI occurring at follow-up. However, recent studies indicate that higher HDL levels are associated with a lower risk of MCI and dementia. Possible explanations for the different results by some researchers include the time from the measurement of HDL levels in the context of cognitive function assessment. It has been suggested that cholesterol levels decline before the development of dementia, and therefore, measurements late in life or during illness may be subject to error [18].

Our results also highlight the impact of anemia on cognitive function. Patients with lower red blood cell parameters scored significantly lower on the MMSE. Patients with ACS are at an increased risk of blood loss and anemia due to their antiplatelet and anticoagulant treatment. Anemia is one of the reversible risk factors for cognitive dysfunction. By ensuring that patients have regular morphology assessments, we can detect and treat it relatively easily and therefore negate its impact on neurocognitive function [19].

Age is considered one of the main risk factors for both MI, CI and depressive disorders [20,21]. Also in our study, we observed that people with CI are significantly older compared to those without deficits. It is important to note that with aging, there is some physiological decline in cognitive functioning, which should be distinguished from pathological situations. In the case of ‘physiological aging’, the CI that occurs does not worsen but remains stable and does not affect daily functioning. Unfortunately, also among medical practitioners, there is a misconception that CI is normal and inevitable with age. Therefore, recognizing older age as the main cause of the occurring deficits, they rarely undertake further assessment of patients using validated neurocognitive tests. This results in the diagnosis of CI only at an advanced stage, whereas the best chance of improving cognitive status and detecting reversible causes of impairment is when the impairment is minor. 

The latest cardiovascular prevention guidelines point out the association between mental disorders and the development of CVD. They emphasize the importance of screening especially for depression and insomnia in this group of patients [11]. In our project, the high prevalence of peri-infarct mental and emotional disorders is also noteworthy, with the predominance of insomnia found in about 50% of patients. A tendency toward aggression was noted in 40% of the patients, and about 30% presented depressive symptoms. Interestingly, after a 6-month follow-up period, significantly fewer symptoms of insomnia and depression were found but not of the tendency to aggression. Available studies indicate an association of hostility, anger and aggressiveness not only with higher CVD risk but also with higher CVD mortality [22,23]. Perhaps this particular group of patients with a greater tendency toward aggressiveness should be subject to earlier psychological intervention to improve CVD outcomes. More studies investigating the association between CVD and psychiatric disorders are needed.

An even less understood aspect is the impact of mental disorders, including emotional disorders, on cognitive function. For depression, the DSM-V lists cognitive impairment as one of the diagnostic criteria. There is considerable overlap between the symptoms of dementia and depression, while the relationship between them is still unclear [24]. Our results indicate an impact of depressive disorders on cognitive processes, which was reflected by higher Beck scale scores for those with peri-infarct CI. Also, more severe insomnia and aggression were associated with higher CI. In the case of aggression, however, this relationship was only observed among women. This is consistent with the results of Margari et al. who also showed an association of aggression with greater cognitive impairment [25]. Surprisingly, this tendency is only observed in the female group. Most studies link aggression to male gender [26], while studies assessing the impact of gender and aggression in the context of the occurrence of cognitive impairment are lacking.

Life expectancy is increasing, but the quality of life is increasingly important to us. CI significantly impairs the daily functioning of those it affects and those who care for them. It is important to proactively detect it at an early stage and try to modify potentially reversible risk factors for cognitive deficits. This will allow better planning of patients’ care and early referral to appropriate specialists. 

This paper is the first part of a larger project on cognitive function in post-MI patients. In the next phase, it is planned to assess the types of impaired cognitive domains, and an attempt will be made to develop a simple diagnostic tool that could easily and quickly identify those at risk of CI in the cardiac patient population.

## 5. Limitations of the Study

This study has several limitations. First is the relatively large number of patients who did not complete the follow-up. Of the original 468 patients, 364 were eventually assessed twice. We think that this had to do with the outbreak of the COVID-19 pandemic that occurred during the project. 

Another limitation is that those included in our project represent a younger population than the average MI patient (59.09 (±9.64) vs. 63 for men and 74 for women) [27]. This is related to older patients refusing to complete the questionnaire as they were unable to complete it themselves. It can, therefore, be assumed that the prevalence of cognitive deficits in post-MI patients is underestimated, indicating their greater prevalence in clinical practice.

Despite our attempt to approach the issue of CI as comprehensively as possible, we are aware of the existence of parameters that were not analyzed in our study and that affect cognitive function, i.e., pharmacotherapy or peri-infarct rhythm disturbances. This could be a starting point for further research, including by our team. 

## 6. Conclusions

There is a high prevalence of CI and other non-cognitive mental disorders, such as depression, sleep disorders and a tendency to aggression, among post-MI patients. The analysis of the collected material indicates a significant impact of worse cardiac function expressed as EF and BNP, greater severity of coronary atherosclerosis expressed by SYNTAX results and red blood cell parameters and LDL levels on the occurrence of CI in the post-MI patient population. Our results indicate an impact of depressive disorders on cognitive processes. Also, more severe insomnia and aggression were associated with higher CI.

## Figures and Tables

**Table 1:** Baseline characteristics and comparison of participants with and without cognitive impairment for the first MI-related hospitalization detected by Mini-Mental State Examination. Normal distribution for continuous variables was tested using the Shapiro–Wilk test. For normal variables, mean (DS) is reported. Otherwise, median (IQR calculated as Q1–Q3) is presented.

Variables	Without Cognitive Impairment (n = 94)	Cognitive Impairment (n = 74)	*p*-Value	Total (n = 468)
Age (years)	58.3 ± 9.3	60.4 ± 10.2	0.03 *	59 ± 9.6
BMI (kg/m^2^)	27.6 (25.1–31.2)	28.1 (24.9–31.2)	0.82	27.8 (25–31.2)
Clinical Factors, n (%)				
History of diabetes	53 (18%)	42 (24%)	0.14	95 (20%)
History of heart failure	23 (8%)	23 (13%)	0.08	46 (10%)
History of hypertension	173 (59%)	100 (57%)	0.85	273 (58%)
History of myocardial infarction	27 (9%)	29 (17%)	0.02 *	56 (12%)
ACS type, n (%)				
STEMI	182 (62%)	99 (58%)	0.41	281 (60%)
NSTEMI	110 (37%)	73 (42%)	0.33	183 (39%)
SYNTAX (points)	9 (6–14)	10 (7–16)	0.05 *	9 (6–15)
Laboratory Values				
Hgb (g/dL)	13.9 (13.1–14.7)	13.8 (12.7–14.7)	0.23	13.8 (12.9–14.7)
Hct (%)	41 ± 3.8	40.3 ± 4.2	0.08	40.7 ± 3.9
RBC (mln/µL)	4.57 (4.27–4.92)	4.56 (4.2–4.85)	0.31	4.56 (4.26–4.89)
WBC (tys/µL)	9.5 (7.6–11.7)	9.6 (7.9–11.7)	0.36	9.5 (7.6–11.7)
PLT (tys/µL)	229 (194–275.8)	221 (191.3–259.8)	0.21	226.5 (193–272)
Na (mmol/L)	140 (139–142)	141 (139–142)	0.41	140 (139–142)
K (mmol/L)	4.29 (4.02–4.51)	4.25 (4–4.5)	0.53	4.28 (4.02–4.51)
Creatinine (µmol/L)	80 (66–90)	79 (70–91)	0.26	80 (68–91)
Urea (mmol/L)	5 (4.3–6.3)	5.3 (4.2–6.3)	0.78	5.1 (4.3–6.3)
Uric acid (µmol/L)	337 ± 81.6	335 ± 79	0.89	335 ± 80.5
GFR (mL/min/1.73 m^2^)	90 (76–105)	85 (73–101)	0.02 *	88 (75–103)
TN (ng/L)	519 (75.5–3041)	635 (66–4504)	0.56	549.5 (71.8–3434.5)
BNP (pg/ml)	96.9 (53.2–199.8)	127.4 (73.1–265.1)	0.01 *	109.1 (55–214.4)
HDL (mmol/L)	1.1 (1–1.4)	1.2 (0.9–1.4)	0.47	1.1 (1–1.4)
LDL (mmol/L)	3 (2.3–3.8)	3.2 (2.3–3.9)	0.49	3.1 (2.3–3.85)
TG (mmol/L)	1.45 (1.1–2)	1.6 (1.1–2)	0.44	1.5 (1.1–2)
TSH (µIU/mL)	1.1 (0.7–1.8)	1 (0.6–1.7)	0.45	1.1 (0.7–1.7)
CK (IU/L)	51 (25–117)	62.5 (25–156.5)	0.22	52 (25–132.5)
ALAT (U/L)	35 (25–50.5)	35 (25–56)	0.57	35 (25–52)
EF (%)	48 (45–54)	48 (41–50)	0.05 *	48 (45–52)
Psychiatric scale				
Beck’s Depression Inventory	7 (4–13)	9 (6–14)	0.02 *	8 (4–13)
The Overt Aggression Scale Modified	0 (0–1)	0 (0–1)	0.88	0 (0–1)
Insomnia Severity Index	7 (3–11)	9 (4–14)	<0.001 *	7 (3–13)
Athens Insomnia Scale	5 (3–8)	7 (3–10)	0.04 *	6 (3–9)
Epworth Sleepiness Scale	6 (4–9)	6 (4–9)	0.33	6 (4–9)

* The statistical threshold for significance for the *p*-values was 0.05. Abbreviations: ACS—acute coronary syndrome; ALAT—alanine transaminase; BMI—body mass index; BNP—brain natriuretic peptide; CK—creatine kinase; EF—ejection fraction; GFR—glomerular filtration rate; Hct—hematocrit; HDL—high-density lipoprotein; Hgb—hemoglobin; K—potassium; LDL—low-density lipoprotein; MI—myocardial infarction; Na—sodium; PLT—platelet count; RBC—red blood cells; TG—triglycerides; Tn—troponin; TSH—thyroid-stimulating hormone; WBC—white blood cells.

**Table 2:** Characteristics and comparison of participants with and without cognitive impairment for the follow-up visit (after 6 months). Normal distribution for continuous variables was tested using the Shapiro–Wilk test. For normal variables, mean (DS) is reported. Otherwise, median (IQR calculated as Q1–Q3) is presented.

Variables	Without Cognitive Impairment (n = 279)	Cognitive Impairment (n = 91)	*p*-Value	Total (n = 364)
BMI (kg/m^2^)	27.9 (25.3–31.5)	28.1 (24.7–30.9)	0.39	28 (25.2–31.2)
Clinical Factors, n (%)				
History of diabetes	51 (19%)	23 (25%)	0.23	74 (20%)
History of heart failure	24 (9%)	7 (8%)	0.91	31 (9%)
History of hypertension	158 (58%)	57 (63%)	0.5	215 (59%)
History of myocardial infarction	30 (11%)	29 (17%)	1.0	40 (11%)
ACS type, n (%)				
STEMI	170 (63%)	51 (57%)	0.39	221 (61%)
NSTEMI	110 (37%)	38 (42%)	0.46	139 (38%)
SYNTAX (points)	10 (7–14)	9 (6–14)	0.52	9 (6–14)
Laboratory Values				
Hgb (g/dL)	14.6 (13.7–15.2)	14.2 (13.4–15.2)	0.06	14.5 (13.6–15.2)
Hct (%)	43.1 ± 3.5	42.3 ± 3.3	0.02 *	43 ± 3.4
RBC (mln/µL)	4.8 (4.6–5.1)	4.7 (4.5–5.0)	0.19	4.8 (4.5–5.1)
WBC (tys/µL)	7.4 (6.2–8.8)	7.9 (6.6–9)	0.14	7.5 (6.3–8.8)
PLT (tys/µL)	236.5 (201.3–275.5)	248 (215.5–281)	0.18	237 (205–277)
Na (mmol/L)	141 (139–143)	141 (139–142)	0.54	141 (139–142)
K (mmol/L)	4.6 (4.4–4.8)	4.6 (4.4–4.9)	0.7	4.3 (4.0–4.5)
Creatinine (µmol/L)	82.5 (72–92)	78 (65.5–91)	0.02 *	81 (71–92)
Urea (mmol/L)	5.7 (4.7–6.7)	5.4 (4.5–6.5)	0.37	5.6 (4.7–6.7)
Uric acid (µmol/L)	348 ± 89.2	352 ± 73.4	0.74	349 ± 86.2
GFR (ml/min/1.73 m^2^)	85 (71–98)	85 (71–98)	0.18	86 (72–99)
TN (ng/L)	9 (9–9)	9 (9–10.5)	0.13	9 (9–9)
BNP (pg/mL)	32.7 (16.2–64.9)	40.2 (20.9–81.2)	0.07	36.4 (17.4–66.7)
HDL (mmol/L)	1.2 (1–1.5)	1.4 (1.1–1.55)	0.05 *	1.2 (1.1–1.5)
LDL (mmol/L)	1.8 (1.5–2.3)	2.1 (1.6–2.6)	0.05 *	1.9 (1.5–2.3)
TG (mmol/L)	1.4 (1–1.8)	1.2 (1–1.6)	0.24	1.3 (1–1.8)
TSH (µIU/ml)	1.13 (0.8–1.7)	1.1 (0.7–1.6)	0.67	1.1 (0.8–1.7)
CK (IU/L)	116 (87–166.5)	116 (87–166.5)	0.44	115 (85–167)
ALAT (U/L)	26 (19–35)	25 (18–33)	0.57	26 (19–34)
EF (%)	50 (48–55)	50 (47–56)	0.45	50 (47–55)
Psychiatric scale				
Beck’s Depression Inventory	7 (3–12)	8 (4–13)	0.27	8 (4–12)
The Overt Aggression Scale Modified	0 (0–1)	0 (0–1)	0.9	0 (0–1)
Insomnia Severity Index	6 (2–10)	5.5 (3–10)	0.64	6 (2–10)
Athens Insomnia Scale	5 (3–8)	4 (2–7)	0.14	5 (3–8)
Epworth Sleepiness Scale	6 (4–9)	5 (3–9)	0.38	6 (4–9)

* The statistical threshold for significance for the *p*-values was 0.05. Abbreviations: ACS—acute coronary syndrome; ALAT—alanine transaminase; BMI—body mass index; BNP—brain natriuretic peptide; CK—creatine kinase; EF—ejection fraction; GFR—glomerular filtration rate; Hct—hematocrit; HDL—high-density lipoprotein; Hgb—hemoglobin; K—potassium; LDL—low-density lipoprotein; MI—myocardial infarction; Na—sodium; PLT—platelet count; RBC—red blood cells; TG—triglycerides; Tn—troponin; TSH—thyroid-stimulating hormone; WBC—white blood cells.

**Table 3:** Relations between clinical variables collected from patients and the number of points (severity of cognitive impairment) assessed with the Mini-Mental State Examination. Table presents only those variables for which the test results were significant.

Variables	Regression Coefficient (β)	*p*-Value	95% CI
Hgb	0.21	<0.001	0.09 to 0.32
Hct	0.10	<0.001	0.06 to 0.14
RBC	0.65	<0.001	0.32 to 0.97
WBC	−0.11	<0.001	−0.16 to −0.06
K	0.48	<0.001	0.18 to 0.78
LDL	−0.28	<0.001	−0.41 to −0.15

Abbreviations: CI—cognitive impairment; Hct—hematocrit; Hgb—hemoglobin; K—potassium; LDL—low-density lipoprotein; MMSE—Mini-Mental State Examination; RBC—red blood cells; WBC—white blood cells.

**Table 4:** Prevalence of depression, sleep disorders (insomnia and excessive sleepiness) and aggressive tendencies during the first hospitalization (2–3 days of ACS) and after 6 months.

Psychiatric Scale	First Hospitalization	Follow-Up (After 6 Months)	*p*-Value
Mini-Mental State Examination	37% (N-174)	25% (N-91)	*p* < 0.001 *
Beck’s Depression Inventory	32% (N-135)	24% (N-103)	0.003 *
The Overt Aggression Scale Modified	44% (N-154)	41% (N-141)	0.2
Insomnia Severity Index	48% (N-171)	39% (N-139)	0.002 *
Athens Insomnia Scale	52% (N-187)	44% (N-157)	0.003 *
Epworth Sleepiness Scale	15% (N-55)	14,6% (N-52)	0.7

* Statistical significance was set at *p* = 0.05.

**Table 5:** Relations between intensity of psychiatric assessment collected from patients and the number of points (severity of cognitive impairment) assessed with the Mini-Mental State Examination.

Variables	Group of Patients	Regression Coefficient (β)	*p*-Value	95% CI
Insomnia Severity Index	Total	−0.05	0.001 *	−0.1 to 0.0
	Men	−0.04	0.02 *	−0.1 to 0.0
	Women	−0.06	0.03 *	−0.1 to 0.0
Epworth Sleepiness Scale	Total	−0.01	0.7	−0.1 to 0.0
	Men	−0.01	0.84	−0.1 to 0.0
	Women	−0.02	0.69	−0.1 to 0.1
Athens Insomnia Scale	Total	−0.03	0.15	−0.1 to 0.0
	Men	−0.03	0.31	−0.1 to 0.0
	Women	−0.03	0.43	−0.1 to 0.0
The Overt Aggression Scale Modified	Total	−0.1	0.2	−0.2 to 0.1
	Men	0.0	0.96	−0.2 to 0.2
	Women	−0.28	0.03 *	−0.5 to 0.0
Beck’s Depression Inventory	Total	−0.02	0.13	0.0 to 0.0
	Men	−0.01	0.37	0.0 to 0.0
	Women	−0.02	0.38	−0.1 to 0.0

* Statistical significance was set at *p* = 0.05.

## Data Availability

The data presented in this study are available on request from the corresponding author. The data are not publicly available due to privacy.
